# The Deadly Chytrid Fungus: A Story of an Emerging Pathogen

**DOI:** 10.1371/journal.ppat.1000550

**Published:** 2010-01-29

**Authors:** Erica Bree Rosenblum, Jamie Voyles, Thomas J. Poorten, Jason E. Stajich

**Affiliations:** 1 Department of Biological Sciences, University of Idaho, Moscow, Idaho, United States of America; 2 School of Public Health, Tropical Medicine and Rehabilitation Sciences, James Cook University, Townsville, Queensland, Australia; 3 Department of Plant and Microbial Biology, University of California, Berkeley, California, United States of America; 4 Department of Plant Pathology and Microbiology, University of California, Riverside, California, United States of America; University of California San Francisco, United States of America

Emerging infectious diseases present a great challenge for the health of both humans and wildlife. The increasing prevalence of drug-resistant fungal pathogens in humans [1] and recent outbreaks of novel fungal pathogens in wildlife populations [2] underscore the need to better understand the origins and mechanisms of fungal pathogenicity. One of the most dramatic examples of fungal impacts on vertebrate populations is the effect of the amphibian disease chytridiomycosis, caused by the chytrid fungus *Batrachochytrium dendrobatidis* (*Bd*).

Amphibians around the world are experiencing unprecedented population losses and local extinctions [3]. While there are multiple causes of amphibian declines, many catastrophic die-offs are attributed to *Bd* [4],[5]. The chytrid pathogen has been documented in hundreds of amphibian species, and reports of *Bd*'s impact on additional species and in additional geographic regions are accumulating at an alarming rate (e.g., see http://www.spatialepidemiology.net/bd). *Bd* is a microbial, aquatic fungus with distinct life stages. The motile stage, called a zoospore, swims using a flagellum and initiates the colonization of frog skin. Within the host epidermal cells, a zoospore forms a spherical thallus, which matures and produces new zoospores by dividing asexually, renewing the cycle of infection when zoospores are released to the skin surface (Figure 1). *Bd* is considered an emerging pathogen, discovered and described only a decade ago [6],[7]. Despite intensive ecological study of *Bd* over the last decade, a number of unanswered questions remain. Here we summarize what has been recently learned about this lethal pathogen.

**Figure 1:**
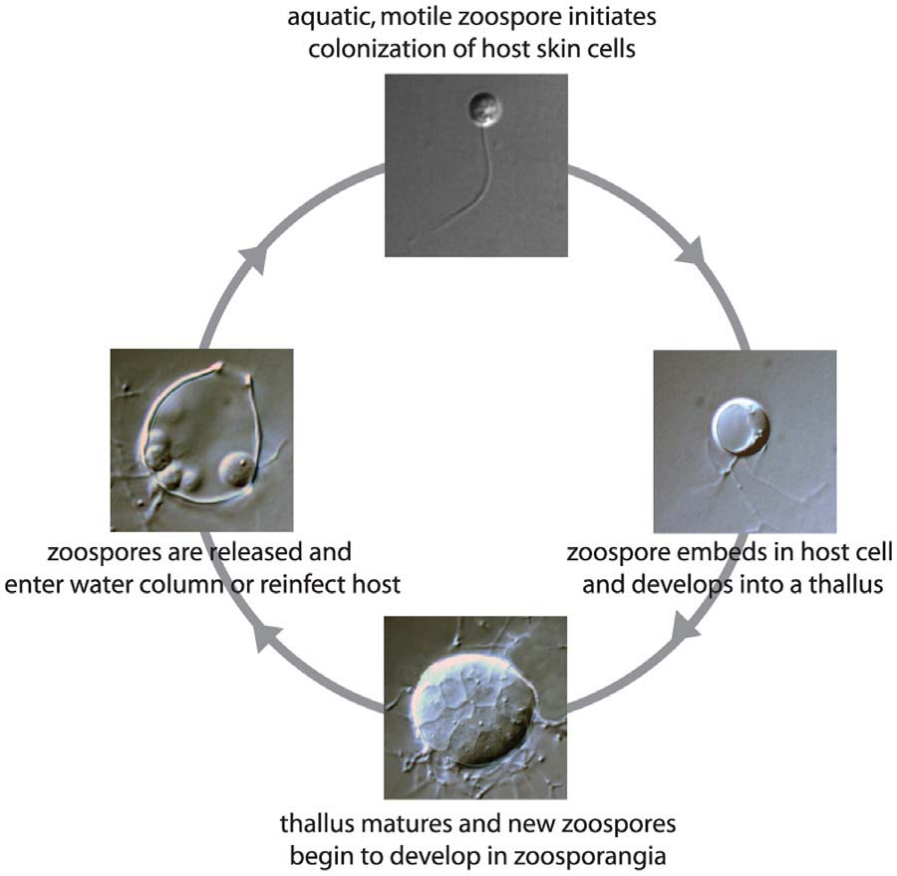
Life cycle of the pathogenic chytrid fungus *Batrachochytrium dendrobatidis*. Images were taken of *Bd* in pure culture grown in 1% tryptone media.

## How Is *Bd* Related to Other Fungi?

*Bd* is a member of a basal group of fungi, the Chytridiomycota, and is the only known member of its order (the Rhizophydiales) to parasitize vertebrates. *Bd* is phylogenetically distant from any of the other ∼1,000 chytrid species [8], and the lack of close relatives capable of parasitizing vertebrates suggests that *Bd* pathogenicity evolved relatively recently. Further, population genetic data on *Bd* isolates collected from different amphibian populations around the world suggest that *Bd* is a recently spread pathogen rather than being endemic with altered relationships with hosts due to environmental change [9],[10].

## How Has *Bd* Spread around the World So Quickly?

Africa was initially proposed as the geographic origin because the earliest evidence of *Bd* is from skin samples from African clawed frogs (*Xenopus laevis*) collected in 1938. African clawed frogs were traded globally for decades (from the 1930s–1960s) for pregnancy assays in humans [11]. Although based on a small sample size, recent population genetic work shows reduced genetic diversity of isolates from African clawed frogs and, instead, high allelic diversity in North American isolates collected from bullfrogs (*Rana catesbeiana*) [10],[12]. Although additional genetic work is needed, these studies suggest that *Bd*'s origin may not be in Africa. Anthropogenic spread of *Bd* is a plausible explanation for at least some introductions [11],[13]. Some amphibian species that are traded globally may serve as disease reservoirs because they can carry *Bd* infections without morbidity. A number of mysteries remain about how *Bd* has dispersed to and persisted in remote pristine environments where anthropogenic introduction is unlikely. If *Bd* can survive independently of amphibian hosts, it must use non-amphibian organic materials as nutrient resources. Although *Bd* DNA has been detected in water bodies [14] and on rocks [15], conclusive evidence of *Bd* persistence in the environment is lacking.

## How Does *Bd* Kill Frogs?

In infected amphibians, *Bd* is found in the cells of the epidermis and pathological abnormalities include a thickening of the outer layer of skin [6]. Cutaneous fungal infections in other vertebrates are not typically lethal, but amphibian skin is unique because it is physiologically active, tightly regulating the exchange of respiratory gases, water, and electrolytes. Thus, the physiological importance of the skin makes amphibians particularly vulnerable to skin infections. It has been hypothesized that *Bd* disrupts normal regulatory functioning of frog skin, and evidence suggests that electrolyte depletion and osmotic imbalance that occurs in amphibians with severe chytridiomycosis are sufficient to cause mortality [16],[17].

## What Factors Are Implicated in *Bd* Pathogenicity/Virulence?

The molecular factors influencing *Bd* pathogenicity and virulence have yet to be conclusively identified. Some evidence suggests that *Bd* enzymatic activity directly influences pathogenesis. The initial penetration of *Bd* into amphibian epidermal cells likely requires digestive enzymes. In culture, *Bd* secretes extracellular proteases that degrade casein and gelatin [18],[19]. At the molecular level, genomic research into *Bd* is revealing intriguing expression patterns in genes such as those for serine protease and fungalysin metallopeptidase [20], two gene families involved in pathogenesis in other fungal pathogens. Full genomes of two *Bd* isolates have recently been sequenced, providing new resources for the study of molecular mechanisms of pathogenicity [21].

## Are There Differences in *Bd* Isolate Virulence?

Several studies have shown variation in virulence among *Bd* isolates. In experimental infections, differences in frog survival have been observed when exposed to different *Bd* isolates (e.g., [22],[23]). Initial proteomic work suggests that *Bd* isolates differ in their proteome profiles [23]. However, controlled infection experiments with reciprocal host isolate treatments and paired genomic and proteomic studies are necessary to identify the functional determinants of *Bd* virulence.

## Do All Frogs Respond Similarly to *Bd*?

Species, populations, and individuals vary widely in susceptibility to chytridiomycosis. Mortality rates in laboratory infection experiments can range from 0% to 100%, depending on the species (e.g., [22],[24]), age of animals [25], and temperature regime [26]. In the wild, some species and populations are extirpated while others, those that survive initial declines, persist with various levels of infection (e.g., [27],[28]). While the disease dynamics are undoubtedly influenced by local environmental conditions, particularly temperature, inherent differences in host susceptibility and behavior are also important. Colonization by *Bd* and subsequent disease development may be influenced by host defense mechanisms, such as secretions of antimicrobial peptides [29] or bacterial commensals with anti-fungal properties [30]. Some species-specific behavioral characteristics such as microhabitat selection, basking, aggregating in retreat sites, or association with water bodies may also affect the likelihood of infection and disease [31],[32].

## How Can We Stem the Tide of *Bd*-Related Declines?

Despite many gaps in our understanding of chytridiomycosis, we are beginning to unravel important elements of this lethal disease and make progress towards amphibian conservation. Multiple conservation strategies have been proposed and are currently being implemented to mitigate the threat of chytridiomycosis. These plans include efforts to limit the spread of the disease, invest in captive breeding programs for highly vulnerable amphibians, and advance basic disease research. Continued research on the biology of both the host and the pathogen is necessary, and efforts to catalog and preserve the *Bd* isolates for ongoing research are particularly important (see http://www.spatialepidemiology.net/bd/ and http://www.bdbank.org/, [33]). The conservation challenges we face with chytridiomycosis—and other emerging pathogens—are best confronted by increasing our knowledge of disease processes from both host and pathogen perspectives.
